# Remarkable Fecundity

**Published:** 1890-07

**Authors:** J. De Leon

**Affiliations:** Ingersoll, Texas


### REMAKABLE FECUNDITY·

I was called to see Mrs. E. T. Page, January 10th, 1890, about 4 o'clock a. m.; found her in labor and at full time, although she assured me that her "time" was six weeks ahead. At 8 o'clock a. m. I delivered her of a girl baby; I found there were triplets, and so informed her. At 11 a. m. I delivered her of the second girl, after having rectified presentation which was singular, face, hands and feet, all presented, I placed her in proper position, and practiced "version." This child was "still-born," and after considerable effort by artificial respiration it breathed and came around "all right." The third girl was born at 11:40 a. m. This was the smallest one of the four. In attempting to take away placenta, to my astonishment I found the feet of another child. At 1 p. m. this one was born; the head of this child got firmly impacted at lower strait, and it was with a great deal of difficulty and much patient effort that it was finally disengaged; it was blocked by a mass of placenta and cords. The first child had its own placenta; the second and third had their placenta; the fourth had also a placenta. They weighed at birth in the aggregate nineteen and a-half pounds without clothing; the first weighed six pounds; second five pounds; third four and a-half pounds; fourth four pounds. In the country, and "backwoods" at that, it was impossible to procure a "wet nurse," so with the little help we could control, and feeding the babies on "Reed & Carnrick's Infant Food," they thrived well. From using all the foods on the market I long since found that the above food possessed some qualities that I failed to find in others. Mrs. Page is a blonde, about 36 years old, has given birth to fourteen children—twins three times before this; one pair by her first husband. She has been married to Page three years, and has had eight children in that time. I have waited on her each time.

Page is an Englishman, small, dark hair, age about 26, weighs about 115 pounds. There was quite an amusing incident occurred when I informed him that his wife would give birth to four children; he fell across the bed by his wife's side, threw his heels away up in the air, clasped his legs with both hands, and with a long wail of despair, cried, "Lord, God, Doctor! what shall I do?"

They are in St. Joseph, Missouri, now, having contracted with Mr. Uffner, of New York, to travel and exhibit themselves in Denver, St. Joseph, Omaha and Nebraska City, then on to Boston, Massachusetts, where they will spend the summer.

The birth of quadruplets is not so remarkable, but that they should live and thrive as these have done, is. In about 375,000 births there are quadruplets, and it is a remarkable fact that they always die. Will some of my brother M. D.'s give us their experience with quadruplets? J. DE LEON, M. D.

—*The Dietetc Gazetlte.* Ingersoll, Texas.

